# Research on the Development of Technologies for the Production of Granulated Activated Carbons Using Various Binders †

**DOI:** 10.3390/ma13225180

**Published:** 2020-11-17

**Authors:** Iwona Skoczko, Remigiusz Guminski

**Affiliations:** Department of Technology in Environmental Engineering, Faculty of Civil and Environmental Science, Bialystok University of Technology, Wiejska 45A, 15-351 Bialystok, Poland; guminskir@grand-activated.pl

**Keywords:** granulated activated carbons (GAC), binders, GAC activation, GAC porosity

## Abstract

Activated carbons (ACs) are processed carbon-rich materials with a highly developed inner surface and significant porosity used for different media treatment in municipal and industrial plants. Activated carbon may be manufactured as powdered activated carbon (PAC), gritty activated carbon (in a form of raw angels grains) or granulated activated carbon (GAC). The production of the GAC is based on carbonaceous raw materials and various types of binders. The carbon mass is mixed with the binder and formed in cylindrical granules. The binder’s recognition is in a scientific literature side-topic and still needs wider examination. For many years GAC production have been concentrated on the possibility of using sodium carboxymethylcellulose (SCMC). Therefore, the aim of the research was to develop a new binder, in the available technology of granulated activated carbon production. Such binders were tested such as: tall oil (TO), sugar beet molasses (SBM), sodium carboxymethylcellulose (SCMC), SCMC using a verified technological process and SCMC with the addition of gas tar (GT). The conducted research shows that all the quality requirements were met by activated carbons with SBM as a binder. Additionally they showed very high adsorption properties. The manufacturing process was shorter in comparison to other tested binders and more efficient.

## 1. Introduction

Activated carbons (ACs) are processed carbon-rich materials with a highly developed inner surface and significant porosity. They play an important role in the processes of liquid and gas treatment in municipal and industrial plants. Carbon adsorbents are commonly used to remove color, taste, organic and inorganic pollutants from water and wastewater [1,2]. Carbon adsorbents have also found application in the processes of air purification from odor-generating plants (i.e., sewage treatment plants) and in pollutants contained in combustion and waste gases [3,4,5]. The market demand for activated carbons of various properties is constantly increasing. The essence of the use of activated carbon is to optimize the process of individual media treatment (water, sewage, gases, air, etc.) taking into account economic and technological conditions [3,4,5]. The most expected is to produce a universal sorbent with very good sorption properties [6]. The production of the activated carbon is based on various materials of organic origin, such as wood, hard and brown coal, polymers and waste from the wood industry, fruit stones and shells, various raw materials and materials of synthetic origin [7,8,9,10,11,12]. Apart from coal raw materials, various types of binders are used to produce activated carbons. As binders, coal tar, methyl cellulose, bentonite, clay, sugars, glycerine, glycol, various oils and many other substances are most often used [7,8,9,10,11,12]. They may also be waste substances from industrial production [13]. The selection of raw materials for the production of activated carbon determines also the typical physical properties of the product, including its mechanical strength and porosity. Cost, availability, grain size and mineral content play an important role in the selection of raw materials [14,15]. The production of activated carbons involves carbonization of the raw material in a dusty or granular form, and then activation of the obtained carbonizate. The activation process is often preceded by mechanical treatment of the raw coal, depending on its natural properties, and on the desired form of the final product. Raw materials from which are obtained carbonization products of low mechanical strength, e.g., brown coal and hard coal dust, must be molded or granulated into appropriate shapes with the use of a binder before carbonization.

One of the most common ways of granulated activated carbons (GACs) formation is the extrusion of the carbon mass mixed with the binder through the specific forms, which results in cylindrical granules. The binder used to GAC production should join individual fragmented particles of the carbonaceous material and give appropriate mechanical strength the newly formed product. It allows for its further processing and gives the granules, after their carbonization, mechanical strength to resist crushing and abrasion. Moreover, it increases their reactivity towards the activating agent [15,16]. During physicochemical activation, free intergranular spaces are created, enabling the formation of a system of capillaries with specific shapes and dimensions. The products of thermochemical processing of hard coal, brown coal and petroleum in the form of tar, pitch or asphalt might be used as binders [16]. Byproducts of the wood and food industry also play an important role in this field. Binders may be recovered from used and processed substrates such as sugar beet molasses, prepared tars from leafy trees, sulphite lye, corn syrup or starch [16,17]. There is not much data to be found in the available literature on the new and improved binders used for the production of granulated activated carbons. Scientists are looking for new AC binder, which may improve AC properties, porosity and mechanical strength. It should be effective in coal-dust-particles binding, economical and ecofriendly. Literature related to the subject matter in recent previous publications mostly informs about different chemical substances used as new binders. Among others, there are mentioned chemical substances as: copolymers of ethylacrylate and xanthan gum [18], mix of hydroxypropyl methylcellulose and ammonium nitrate [19], a humic acid-derived sodium salt, polyvinyl alcohol, a novolac phenolic resin, Teflon, an adhesive cellulose-based binder [20], lignocellulose [21] and polymerized vinylbenzyltrimethyl ammonium alanate [22]. There is little information available about the physical or natural binder as a mixture of carbon black with vinyl acetate polymer, or epoxy polymer, or surface-active substance [23], bentonite binders (containing either iron, zinc or copper cations) [24], colophony resin and carnauba and bee waxes [25]. The least data is on waste substances as grape must [26] or waste from the food industry [27]. Taking into consideration current environment pollution with chemical products new research should focus on natural or waste-based binders. Activated carbon works as an adsorber only for a few years, then is regenerated or neutralized by incineration. During those processes chemical substances release back to the environment and may persist for a long time in air, ground or ice. The best solution is waste material usage as AC binders.

As far as the development of new binders is concerned, since the 1970s most of the activated carbon production plants in the world have concentrated their research mainly on the possibility of using sodium carboxymethylcellulose (SCMC), known as cellulose gum [16]. In the form of a 5% aqueous solution it can be used as a granulating binder of carbon matter. Nevertheless, the carbon final products made on the basis of raw material SCMC did not receive a fully positive assessment in the industrial company chosen for research for this work. Granulated activated carbons formed with sodium carboxymethylcellulose-based binders were not allowed for serial production. A decision to start with experiments on GAC production based on new binders was taken. Therefore, the aim of the research was to develop a new binder, in the available technology of granulated activated carbon production. Liquid waste from local food production plant as a sugar beet molasses (SBM; without chemicals) and gas tar (GT) from hard coal degassing were used. Tests with sodium carboxymethylcellulose were conducted to comparison. Most of the worldwide recovered waste–molasses is used for industrial purposes: for the production of baker’s and brewer’s yeast, alcohol, citric acid or for biogas production, and fertilizer or animal feed. The novel idea of using it as an alternative binder to granulate activated carbon. The new AC production settlings with SBM may give the opportunity to demonstrate that waste substrates are equally valuable binders as chemical commercial offering AC final products better technological parameters.

## 2. Material and Methods

### 2.1. Technological Processes

The aim of the research was to produce granulated activated carbon using various types of granule forming binders. The research was carried out in a carefully selected manufacturing plant that specializes in the production of various activated carbons with different applications. The chosen plant is the leader in the production of activated carbon in Eastern and Central Europe. As part of the conducted research, a binder preparation installation was built and test series of activated carbon were made. The program was divided into the following phases:

- Making model series of granulated activated carbon with the use of selected binding agents,
- Selection of binders with optimal quality parameters,
- Making prototype batches of activated carbons and sorbents with the use of a selected binder and qualitative evaluation of the obtained materials,
- Execution of final products and their evaluation.

The tests schedule led to experiments on each selected binder to make prototype batches of sorbents. The program of tests was as follows:

- Tests with sugar beet molasses (SBM),
- Tests using SCMC test I (SCMC I),
- Tests using SCMC-test II (SCMC II),
- Tests using SCMC + gas tar (SCMC + GT),
- Tests using tall oil (TO).

The following test range was valid for each of the tested binders:

- Production of granulated activated carbon using industrial installations,
- Manufacturing of media,
- Manufacturing of carbon sorbents.

In total, to obtain reliable and repeatable research results, 5 series of tests were carried out with all selected binders. Additionally, tests with the use of SCMC were repeated more times because of poor features of granules manufactured with this binder.

### 2.2. The Binders Preparation Method

An experimental procedures were developed with the following binders: tall oil (TO), sugar beet molasses (SBM), sodium carboxymethylcellulose (SCMC), known as cellulose gum—5% solution using a verified technological process, and sodium carboxymethylcellulose (SCMC) with the addition of gas tar (GT). SCMC and TO were bought as a commercial products with chemical features required for granulated activated carbon (GAC) production. Gas tar was obtained at a temperature of 1000 °C while degassing hard coal in the analyzed activated carbons manufacturing plant site. SBM was post-production waste given form sugar industry plant for testing. Binders were necessary to granule formation and GAC production.

#### 2.2.1. Sugar Beet Molasses (SBM)

The sugar beet molasses raw material meeting the requirements of PN-76/R-64772 was purchased in the sugar manufacturing company. The molasses was stored in carbide drums with a capacity of 2 Mg, and then heated in a chamber dryer and poured successively into a binder tank. From the tank the product was dosed with a pump to the paste mixer. The used molasses had a viscosity of 500–540 mPa/s (at 70 °C) and a coking rate of 8–12%. Moreover, the average sample contained: 10% of moisture, 4.86% of ash, had a density of 1.3739 g/cm^3^ and pH = 8.33.

#### 2.2.2. SCMC

In the I and II trial was used technical SCMC corresponding to the requirements of BN-75/6069-05. The raw material contained: 12.19% of moisture, 19.8% of chloride and had pH 11.5. Aqueous solutions of SCMC were prepared in an industrial SCMC installation located on the industrial in the chosen production plant. There was used solution at a concentration of 5.1–6.0% and a viscosity of 41–59 mPa/s (at 70 °C).

#### 2.2.3. SCMC + Gas Tar (SCMC + GT)

The tested binder contained 85% of SCMC and 15% of gas tar. The tested technical SCMC corresponding to the requirements of BN-75/6069-05 is described above, while the gas tar is regulated by the requirements of PN-78/C-97036. It had a density of 1.1915 g/cm^3^, coking number of 26.85% and viscosity of 45.7 mPa/s (at 70 °C). Aqueous solutions of SCMC were prepared in an industrial SCMC installation located in the chosen production plant. An aqueous SCMC solution was dosed to the high-speed mixer with one pump and gas tar with the other pomp.

#### 2.2.4. Tall Oil (TO)

For the research there was used tall oil conforming to BN-86/7313-02 standard. The tall oil stored in carbide drums was heated in a chamber dryer and after liquefaction was poured in portions into the binder tank. The tall oil had a density of 1.0219 g/cm^3^, coking number of 0.82% and viscosity of 70 °C—29.4 mPa/s.

### 2.3. Individual Technological Process Phases of GAC Production Using the Tested Binders

Experimental procedures for this work included granulated activated carbon production from hard coal and different binders. The parameters of individual stages of the technological process determined during model batches with the use of the tested binders are listed in Table 1, Table 2 and Table 3.

The activated carbon production process included such unit processes as:

- Crushing and grinding of raw hard coal,
- Production of paste from binders and coal dust,
- Granulation,
- Drying and surface hardening of granules,
- Carbonization,
- Activation.

#### 2.3.1. Crushing and Grinding of Raw Hard Coal

Coal was crushed to a dusty form with a granulation of about 60 microns in the stone mill (Figure S1). The hard coal was supplied from polish hard coal mine as material inferior in terms of quality: waste of coal mining. The hard coal’s parameters were: sinterability of 75–85 Recyclability Index (RI), granulation of 8–50 mm, moisture content maximum of 4% and ash content maximum of 3.5%. The used for research stone mill was cylindrical-shaped and its volume was 1 m^3^. Dust productivity was about 300 kg/h.

#### 2.3.2. Coal-Binder Paste Production

After grinding received hard coal dust was mixed with tested binders (Section 2.2) in appropriate proportions presented in the Table 1. The process concerned coal-binder paste production (Figure S2). Beside basic substrates (coal; dust and binder) to the paste was added NaCO_3_ solution with capacity and concentration given in the Table 1. The paste was prepared in the mixer with horizontal axis mixers. Paste productivity was about 300 kg/h. The binders were a mixer from an intermediate tank heated with waste technological steam. The average temperature of molasses dosed to the mixer was 55–65 °C.

#### 2.3.3. Granulation

The coal-binder paste was fed to the granulator, in which the so-called raw granules were formed. Raw granules productivity is presented in the Table 1. Granulation was carried out in factory industrial granulators and technical properties of raw and dried granules are presented in the Section 3 and their appearance in the Figure S3. The load of the granulator was 300–320 kg of carbon mass per hour. The charge of the granulator was 40–45 amperes.

#### 2.3.4. Drying and Granules Surface Hardening

From mixer granules were transferred then to rotary dryers heated by gas fumes. In dryers, the granules were dried and their surface hardened. Drying of raw granules was realized at the temperature in the charging part between 400 and 420 °C and between 280 and 300 °C in the discharging part. Raw granules stayed in dryers for about 20–25 min. Maximum capacity of one dryer was 1000–1200 kg/h. Granulation times differ for every binder and were as follows:

- SBM—9 h of granulation,
- SCMC I—20 h of granulation,
- SCMC II—11 h of granulation,
- SCMC + gas tar—9 h of granulation,
- Tall oil—11 h of granulation.

#### 2.3.5. Carbonization

The dried granules were then directed to a special rotary countercurrent carbonization furnaces for degassing. The temperature was regulated there by control of the gas and air supply to the furnace. To make the furnace hot, the heat of vapor gas burning produced during the carbonization was used. The furnace rotation time was 35 s. Technological parameters of drying and carbonization are shown in the Table 2. The optimal charging of the carbonization furnace was 600 kg/h of dried granules.

#### 2.3.6. GAC Activation

Granule activation is the last process of GAC manufacturing. Technological parameters of activation are given in the Table 3. Activation run in the rotary furnaces (Figure S4) at the temperature of 820–1040 °C. The basic activator was steam-gas. In the experimental process granules were activated using also waste carbon dioxide recovered after the carbonization process. CO_2_ was also recovered from the activation process itself. Activation furnaces’ productivity is presented in Table 3. The activation process was not realized in one single batch. Experiments were conducted with 3–4 series of activation. The number of process’ reduplications depended on final GAC porosity. Individual charcoal materials with tested binders required following activation times:

- SBM test—3 series of activation—total activation time 17 h,
- SCMC I—3 series of activation—total activation time 47 h,
- SCMC II—3 series of activation—total activation time 68 h,
- SCMC + GT—3 series of activation—total activation time 47 h,
- TO—4 series of activation—total activation time 68 h.

Activated granules had a cylindrical shape, diameter about 1.4 mm and length 10–20 mm. Final GAC formed in this way was directed to a sifter in order to obtain the appropriate grain fractions and delate excessive ash content. Additionally during the production process of GAC, high-calorific steam-gases were produced. They were discharged to the furnace of the recovery boiler producing steam for technological and economic purposes.

### 2.4. Test Methodology

Within the framework of the research the parameters of activated carbon and the tested binders were conducted in accordance with the applicable standards presented in Table 4. Parameters measured during experiments are described in Table S1 (Supplementary Information).

## 3. Results and Discussion

The paper present the results of the research carried out on raw materials, semifinished and final GAC products from individual technological process stages. The conducted tests led to the production of granulated activated carbon. It is active carbon (AC), formed with a cylindrical shape, diameter about 2 mm and length 10–20 mm. It is designed for water treatment, both in large water supply stations and in small filter and container installations. The experimental production trend to form the new product in place of the previously produced and popular older equivalent. There was estimated that the new GAC due to its high specific surface area and developed pore structure may be highly effective in removing organic contaminants, pesticides, detergents and a number of micro pollutants harmful to health from water. It could be also used to water dichlorination and its taste and smell improvement.

### 3.1. Crushing and Grinding Research

One of the first stages of activated carbon production was crushing and grinding of the raw material. The properties of coal dust obtained during grinding of hard coal are presented in Table 5.

Dust tests were carried out at each stage of the research. Dust showed a fairly constant ash content and moisture content. The apparent surface area of the dust ranged from about 0.2 to 0.35 m^2^/g. The grain diameter was kept within 20 µm in order to obtain as many grains as possible under 0.088 mm, which can be used in the next production step. Measured parameters presented in Table 5 were conducted using methods given in Table 4. Obtained results differed in every experimental series. The Table 5 shows average values from five conducted experimental series.

### 3.2. Research on Raw and Dried Granules Formation

The granules were formed from carbon paste with the parameters listed in Table 1. The homogeneous paste was fed via the granulator so that all particles were of equal size. The optimum moisture content given in Table 6 allowed for the formation of equal rolls of about 2 mm diameter. It should be noted that the use of molasses made it possible to maintain lower moisture content than that of the SCMC. This value ranged from 9 to 11% for molasses to 17 to 20% for the SCMC. Forming raw granules with the use of tall oil required the lowest moisture content, i.e., about 3–4%. At all stages the granules were characterized by a similar bulk density and ash content. In conducting research, it was found that the paste with all binders granulated correctly, without cracking or breaking the granules. The share of individual binders in the paste ranged from 20 to 24%.

In the SCMC I test and in the SCMC + GT test, as a result of an emergency situation, drying was carried out with insufficient heat (190–220 °C). The required temperatures were 280–300 °C. during experiments granules with the SBM binder and in the SCMC II test were dried at 200–300 °C and with TO at 260–420 °C. In terms of operating parameters, the water solution of the SCMC as a binder had the greatest advantages (Table 6). The production allowed one to obtain dry granules after the process within 90% of the input mass. According to the standards, dried granules should be resistant to crushing in the hand, without visible water evaporation and smell of thermal decomposition products of organic substances. Their characteristics are presented in Table 6.

All the binders allowed one to achieve a similar diameter of granules, i.e., 1.85–1.94 mm, which means that no losses of the binder or carbon base material were recorded during production. The ash content was also at a similar level of 600 g/dm^3^ and the volatile matter content of 30%. The lowest moisture content was found in the second test with 0.11% SCMC and 0.16% tall oil, while the remaining binders kept the moisture content within 0.9%.

### 3.3. Experiments with Carbonization

The next process of activated carbon production after granulation and drying was carbonization. The detailed characteristics of individual samples after carbonization are presented in Table 7.

Each series of produced carbonizate was tested. According to the standards, the bulk mass of carbonized granules should be kept at a level of 720 ± 30 g/dm^3^, maximal content of volatile parts should not exceed 10–12%. Granules mechanical strength ought to reach 99.5%. It was required to create dry granules with a diameter about 1.4 ± 0.1 mm. These parameters were not maintained for every tested binder, as shown in Table 7. The tested binders affected the properties of the granulate in different ways. The granules formed with the use of SCMC had the lowest physical parameters, i.e., mechanical strength—78% and abrasion resistance—0.1–0.2%, which disqualified them as a final product. Granules with tall oil had high strength, but they were characterized by significant contraction after drying above 10%. This change means that the product will not be stable when used on the market.

The temperature in the carbonization furnaces was between 270 at the charging and 650 °C at the discharging. Exceeding these temperatures in the feeding channels could lead to the furnace failure. It is also responsible for defects of the carbonized granules. What is more, if granules are injected into the furnace in an undried form, it may cause their deformation and roughness. Similar defects might appear if the temperature of the charging process is too high. Unfortunately, the temperature increased during the carbonization furnace filling, which resulted in the formation of abnormal charcoal. This phenomenon was observed about 3–4 h after the lower loading in the first series of experiments. To solve this problem in next series temperature growth of the granules was controlled to a maximum of 6 °C per 1 min of their retention time in the furnace. In this way, the burning of the granules was prevented. It helped also avoid excessive lowering of the final temperature of the carbonization. The granules were not deformed nor cracked. These defects occur when too high moisture content after the previous processes run from the drying and granulating process. During the first SCMC test it was not possible to maintain the required process temperature conditions. A decision was made to make an additional model series with the use of SCMC’s second test. In the case of the test SCMC and gas tar, despite the insufficient temperature conditions for drying and carbonization, the need for an additional test was not taken into account, because in spite of properly conducted laboratory drying, carbonization and activation processes also negative results were obtained. The granules were unstable, glue and easily crushed in the hand.

In the course of the research it was noticed that in the previous application of wood tar as a binder, there are practically no difficulties in carbonization consisting in agglomeration, i.e., blending into smaller and larger lumps of carbonized granules. As writes Januszewicz and his team [9] carbonization is an important and difficult stage of the technological process. During carbonization, coal changes its state of plasticity. The correctness of those changes also depends on the type of the used binder. When changing the binder, the described difficulties may significantly intensify or even make it impossible to conduct the process and disqualify the binder, so the process should be conducted carefully and each stage should be observed, which emboldened Carvalho’s group [5]. Conducting our own examination, when using SCMC based binders, caking of carbonizates has often been noted. Additionally, this time, under proper temperature conditions (600–720 °C), the second SCMC test was obtained in the form of glues. Ballias and Reimert [28] and Benaddi’s team [29] also confirm that the degree of carbonization of the organic raw material has the most significant impact on the pore volume, specific surface area and pore size distribution of activation products.

**Figure 1:**
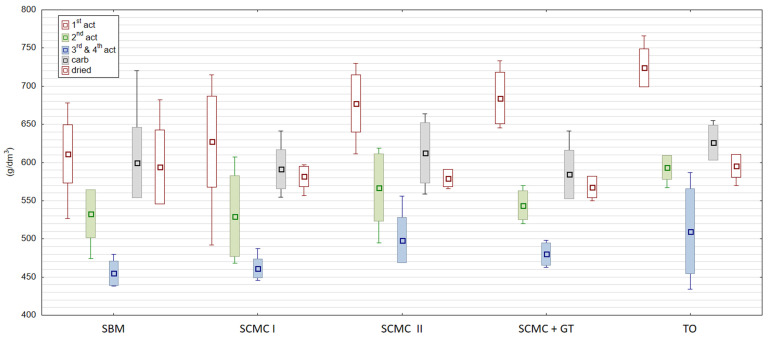
Bulk mass of activated carbons after individual production series (g/dm^3^). Source: own elaboration. 1st act—first activation series, 2nd act—second activation series, 3rd and 4th act—third and fourth activation series, carb—carbonization, dried—granules drying.

**Figure 2:**
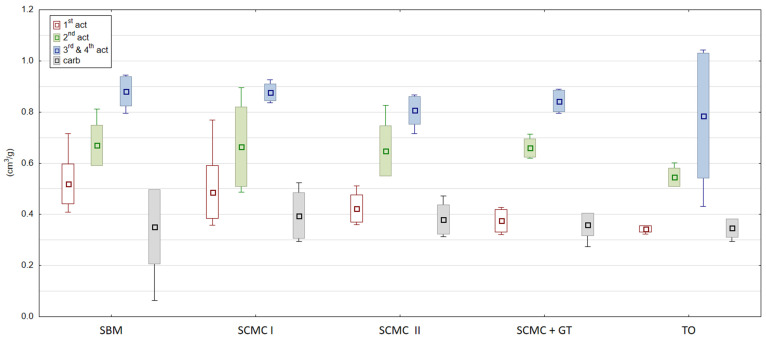
Water absorption of activated carbons after individual production series (cm^3^/g). Source: own elaboration. 1st act—first activation series, 2nd act—second activation series, 3rd and 4th act—third and fourth activation series, carb—carbonization.

The aim of the carbonization process was thermal decomposition of raw materials (coal, binders and additional) and should eliminate other than carbon elements (oxides, hydrides and nitrates) creating porous mass structure [30]. That is why it was important to maintain required process conditions given in Table 2. Deficiency of the charcoal gas was the reason for insufficient temperature conditions of granule carbonization in the 1st SCMC test and in the 2nd SCMC + gas tar test. Granules with the SBM binder were carbonized carefully, i.e., at 460–540 °C, while those with tall oil were carbonized at 550–660 °C, regulating the temperature so that the granules did not stick together and were sufficiently carbonized. In the latter two tests, there were no difficulties in agglomerating the granules. As a result of the carbonization process, the content of volatile parts in dried granules decreased from 30–33% to 8.5–9.0%. Proper thermal conditions were ensured in the second SCMC examination and in the test with tall oil. The rest of the carbonizates had this parameter about 18.2% for the molasses test, 20.1% for the first SCMC test and 26.4% for the SCMC + gas tar. The average samples of carbonizates produced with the use of various binders (Figure 1) were characterized by a bulk mass of 590–650 g/dm^3^ and water absorption of 0.30–0.38 cm^3^/g (Figure 2). It was also observed that granules using SBM as binders are very reactive and after thermal treatment processes (carbonization and activation) they should be cooled without air, otherwise they glow and incinerate. Lee and others [7] discovered and Cal’s team [31] proved that the thermal effect and the heating time make up the carbonization efficiency, but the process does not considerably modify the properties of the charred texture.

The porosity, which is the most important feature of activated carbons, reached the highest value for granules with molasses (total pores volume of 0.36 cm^3^/g). GAC manufactured using SBM showed the greatest pores volume with Ø 7500–1500 nm as 0.22 cm^3^/g in comparison to other binders. This parameter hesitated from 0.18 for TO to 0.13 cm^3^/g for SCMC I. The lowest porosity was observed for granules with tall oil. Pores that are formed during carbonization are, in fact, very small and sometimes they are partially blocked by messy coal ash. For this reason, the pores in the raw coal material need to be additionally expanded and hardened by a special thermal modification in order to be activated [32].

### 3.4. GAC Activation Tests

**Figure 3:**
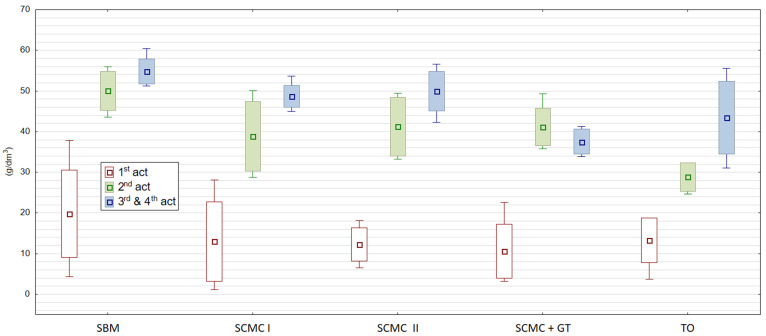
Dynamic activity in relation to C_6_H_6_ of activated carbons after individual (1, 2, 3 and 4) activation series (g/dm^3^). Source: own elaboration. 1st act—first activation series, 2nd act—second activation series, 3rd and 4th act—third and fourth activation series, carb—carbonization.

The essence of the process of active coals (ACs) production is the activation of carbonized charcoals. It opens the internal pores in the coal structure and creates a large sorption surface. Activation within this work was carried out 4 times. Figure 1, Figure 2 and Figure 3 show the properties of individual granule samples after each activation stage, while Table 8 and Figure S4 show the properties of averaged samples after each activation stage.

Figure 1, Figure 2 and Figure 3 show the properties of individual samples taken during activation series I, II, III and IV. Several degrees of activation are possible when GAC production is based on new raw material and better features of produced activated coal are expected. The chosen for the experiments manufacturing plant wanted to change old binder–wood tar. Using old binder for GAC production there was reached surface area max. 670 m^2^/g. In conducted research this level of surface area was reached with our tested coals after 3rd and 4th activation for SBM (Table 8). That is why repetition of the activation process was needed many times. Tests showed us that the single activation process was not enough. After 1st activation there was an obtained surface area only of 300 m^2^/g. Decision about 2nd activation was taken and the surface area increased to about 500 m^2^/g, which was not enough as well. The solution was 4-times activation. The GAC with SBM reached the surface area almost 700 m^2^/g of what was comparative to commercial products. Other tested binders did not achieve such a level of surface area. This parameter changed from about 160 to about 650 m^2^/g for SCMC I, from about 117 to 630 m^2^/g for SCMC II, from about 150 to 570 m^2^/g for SCMC+GT and from about 114 to 430 m^2^/g for TO.

In order not to burn granules at the beginning, preventive activation is carried out with the lowest temperature range. Once the raw material is known, single-stage activation can be set using the optimal temperature ranges. In addition, by setting milder temperature conditions in several activation series, a more accurate required pore structure in activated coal is developed. Such a procedure also gives an answer if an additional activator is needed with the binder used, e.g., sodium and potassium. Obtained results in activation process are presented in Figure 1, Figure 2 and Figure 3 and Table 8. For each binder, activation was carried out to obtain at least 0.8 cm^3^/g of water absorption for every processed activated carbon. Among the charcoals activated within the framework of this work, the samples with tall oil were the most reactive. Similar properties have those obtained within the SCMC II. However, they required the longest activation time in production. Our own research showed the SBM carbonizate was activated in lower than standard temperature conditions, i.e., 820–940 °C, while the activation of the remaining granules was carried out at about 100 °C higher temperature, i.e., 960–1010 °C (Table 2 and Table 3). Only during the first activation of the granules from the first SCMC test and the SCMC + TG test, the temperature was lowered to 720–860 °C. The reason to decrease the temperature during process was blending of granules during activation. Such phenomena might appear when activation time is shorter and temperature higher. Granules after carbonization are supplied to the activation furnace as loose mass with residual moisture. It could lead to their caking and clumping. Proper temperature regulation and sintering time need to be under control [33].

All of manufactured GACs required additional purification due to their elevated particle size with a fine grain. Excessive ash content needed to be removed. Parameters of final GAC products manufactured using different binders are presented in Table 9.

After the activation process a big number of lower particles and a high content of undersized grains was distinguished by the following tests: with tall oil, the SCMC II test and the SCMC test with the addition of gas tar. To obtain low-ash-activated-carbon, an acid demineralization process was performed. The activated carbon is poured into acid pickling baths. At the bottom of the bathtubs there is a drainage system enabling the injection of air in the form of microbubbles. Hydrochloric acid solution of about 5% concentration is dosed into the chambers and aeration is activated. Its purpose is to enable demineralization in the whole volume of the tanks. The air bubbles lift the carbon grains, preventing them from falling to the bottom. Demineralized coal soaks up the HCl solution and changes its weight, which accelerates sedimentation. The lack of mixing would lead to a reaction that would be only superficial. The demineralization process lasts a minimum of 8 h, maximum 16 h and depends on the coal parameters that are planned to be achieved. The bathtubs were installed outside the production hall in unheated rooms. When the ambient temperature dropped below 10 °C, the air was replaced by steam at 120 °C. After HCl digestion, the carbon rinsing phase took place, usually carried out three times, until the pH 6–7 was reached. Acidic digestion of active carbon allows for effective elimination of ashes and raising the iodine number. The mechanical strength remained at the level obtained after the activation process, but the value of the bulk density decreased. In the conducted research ash content decreased significantly. This parameter exceeded 17% for GAC with SBM after the activation process and due to acid treatment it reached less than 4%. Active carbons with other binders also showed improved properties. The least ash content was noted for GAC with SCMC. Demineralization with HCl improved another GAC’s features too. It was observed bulk mass decrease because of acid digestion of residual matter reacted with HCl. The process allowed one to open pores and increase surface area of GAC.

**Figure 4:**
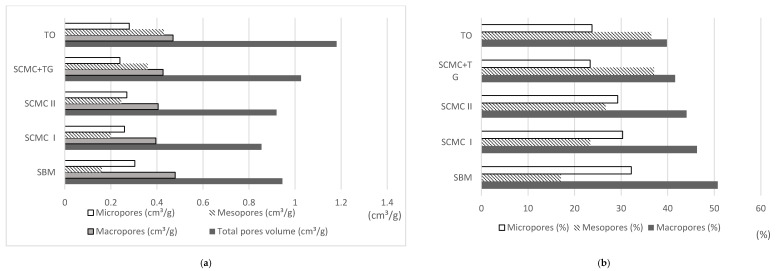
Structural properties of average granulated activated carbon (GAC) samples manufactured using the tested binders. Source: own elaboration, (**a**) micro-, meso- and macropores and total pores volume per 1 g of AC (**b**) micro-, meso- and macropores ratio in AC sample in (%).

According to Sweetman and others [8] the efficiency of the adsorption process depends primarily on the parameters of the porous structure of the carbon materials. In general, the practical application of activated carbons required these adsorbents to have a high volume of fine pores [34]. The adsorption process in micropores took place due to their volumetric packing [16]. The specific surface area of the micropores determined the adsorption capacity of the adsorbate to a significant extent. Mesopores, the so-called transition pores, are characterized by the retention of adsorbent in their channels. Due to their size, what tested Bernal’s team [6] they contribute considerably to the adsorption of larger particle sizes such as, for example, dyes or humid acids. Mesopores act as pathways for the adsorbent particles to move to the micropores. Macropores, on the other hand, are generally not important for the adsorption process with activated carbon. This is due to their very small share in the total adsorbent surface area. They act as transport channels for adsorbent to micropores and mesopores [11,16,35]. On the basis of the conducted research it was noticed that the charcoals containing the most mesopores were produced on the basis of tall oil and a mixture of SCMC with gas tar, whereas the charcoal containing the least amount of mesopores is the sorbent produced on the basis of molasses (Figure 4). Micropores in the highest proportion are found in coal formed with molasses and then with tall oil. However, their presence is minimal in coal with SCMC. Most macropores are found in active carbon (AC) produced on the basis of molasses, and the least in those produced with tall oil. The porous structure is comparable for two series of coal with SCMC I and SCMC II. The largest number of pores in total was found in tests with tall oil, SCMC in a mixture with gas tar and molasses. The smallest number of total pores was observed for tests with SCMC I and SCMC II (0.8543 and 0.9194 cm^3^/g respectively). Due to the strong relationship between the porous structure and the adsorption efficiency of the different adsorbates, the selection of the appropriate activated carbon, in terms of its specific surface area and dominant pore size, should be correlated with the properties of the removed molecules from different media (water, wastewater and gas) [6,13,17].

## 4. Summary

Conducted research on GAC creation using different binders allowed us to find such a binder, which may be approved in commercial production. Substrates cost, their availability, grain size and mineral content are important indicators in the selection of binding materials. Proper binder should join coal dust particles and give appropriate mechanical strength the newly formed product [36]. It allows for GAC’s processing (granulation, drying, carbonization, activation and acid treatment) and is responsible for required parameters of GAC’s final product. Manufacturing time plays also a substantial role. Total time of production for SBM was over twice shorter than the other binders:

- SBM—drying 0.33 h, granulation—9 h, carbonization—20.66, activation 17 h, acid treatment—8 h, total: 55 h;
- SCMC I—drying 0.34 h, granulation—20 h, carbonization—40.5, activation 47 h, acid treatment—10 h, total: 117.84 h;
- SCMC II—drying 0.33 h, granulation—11 h, carbonization—23.5, activation 68 h, acid treatment—10 h, total: 112.83 h;
- SCMC + GT—drying 0.42 h, granulation—9 h, carbonization—30.5, activation 47 h, acid treatment—12 h, total: 98.92 h;
- TO—drying 0.42 h, granulation—11 h, carbonization—21.83, activation 68 h, acid treatment—16 h, total: 117.25 h.

The last stage of production was drying of active coals, followed by their preparation for sale.

Conducted research and the experimental test proved that sugar beet molasses may become one of the most efficient and less requiring binders in granulated activated carbon production. GAC production with SBM was the shortest and allowed us to obtain the best active carbon properties among other tested binders. In comparison to other examined binders it did not have toxic properties as gas tar. GT in higher temperatures releases during carbonization and activation harmful gases including polycyclic aromatic hydrocarbons, which may cause dangerous air pollution. Therefore, it is important to maintain appropriate conditions for these processes by carefully controlling the temperature. Production of good granulated activated carbon should lead to the creation of product with developed surface area, high porosity, optimal mechanical strength and low ash content.

In the scientific literature [7,8,16,17,36,37] new carbon materials are described when produced without a binder. Activated carbons are prepared in different morphologies, granulation, in a form of pellets or monoliths. Authors tested the formation of coal material without a harmful binder. They replaced it with other chemical substrates or the realized carbonization process slowly changing temperature. Nevertheless in comparison to the commercial product with binders some active carbon features were worse, i.e., surface area, bulk mass or iodine number. Science development in the field of active carbon production is currently unstoppable. Due to serious environment and air pollution industrial plant manufacturing AC should look for natural products being not harmful for ecosystems. GAC production using SBM matches this trend. SBM as a natural product easily removed from the environment may become an expected substrate. Tests based on changing physical conditions that allow one to decrease the amount of binder or realized AC production without any binder will be the aim of the next cooperation between university and the industrial plant, which was involved in the study presented in this paper.

## 5. Conclusions

1. The conducted research shows that all the quality requirements were met by activated carbon produced using sugar beet molasses as a binder. Additionally they showed very high adsorption properties.
2. All requirements were also met by carbon tests with SCMC I. The granulated carbons with the remaining binders were normative, but raised reservations regarding mechanical strength or dynamic activity in relation to ethyl chloride.
3. Normative dynamic activities in relation to benzene have all manufactured coals with the exception of the test with a binder SCMC + GT. The highest index was found for coal with SBM (57 min). All GAC had a dynamic activity in relation to chloropicrin above 120 min. The highest rate of 161 min was for molasses. The tested coals had a normative parameter of dynamic activity in relation to ethyl chloride, but in SCMC + GT and SCMC I tests had lower requirement limits. The highest index of 40.7 min was found for coal with molasses.
4. The specific surface area of the obtained activated carbons ranged from 574 to 693 m^2^/g. In terms of mechanical strength, the manufactured coals formed the following series of increasing values: OT (86.5%), SCMC + GT (89.2%), SCMC I (90%), SCMC II (92.5%) and SBM (95.7%).
5. The abrasiveness of activated carbons did not exceed 1%, only in the tall oil test it was 1.3%.
6. Total production time of GACs was the shortest for charcoals with SBM (47 h). Other tested binder required doubled time of manufacturing to obtain the final product.
7. GACs produced on the basis of SCMC, i.e., SCMC I and SCMC II, were not sufficiently well hardened for the finished product granules. Although their sorption properties were compared to SCMC + GT-based GAC granules, they were disqualified by their low level of mechanical strength and very high abrasion level.

## Figures and Tables

**Table 1:** Raw material consumption and granulation efficiency.

Binder	The Composition of the Paste and the Consumption of Raw Materials						Granulation Efficiency				
	Coal		Binder		Sodium Carbonate Solution		Paste Process	Productivity		Waste	
	kg/h	%	kg/h	%	kg/h	%	kg/h	kg/h	%	kg/h	%
SBM	668	79.0	168.5	19.9	9.5	1.1	846	684	81.8	162	19.2
SCMC I	632	79.4	164	20.6	na	na	796	705	88.6	91	11.4
SCMC II	not tested										
SCMC + GT	583	75.6	157.3 SCMC 30.8 GT	20.4 SCMC 4.0 GT	na	na	771	699	90.7	72	9.3
TO	606	75.4	173	21.5	25.0	3.1	804	690	85.8	114	14.2

na—not applicable; source: own elaboration.

**Table 2:** Technological parameters of drying and carbonization.

Binder	Drying			Carbonization			
	Temperature (°C)		Productivity of Single Series (kg)	Temperature (°C)		Charge (kg/h)	Productivity of Single Series (kg)
	Charge	Discharge		Charge	Discharge		
SBM	520–580	220–280	6240	150–200	460–540	275–300	3672
SCMC I	460–580	190–220	10660	120–200	200–560	250–275	7776
SCMC II	600–630	200–300	6760	200–220	600–720	250–325	3888
SCMC + GT	540–560	180–200	5720	120–200	200–560	125–250	3672
TO	500–650	260–420	5720	200–240	550–660	250–275	2808

Source: own elaboration.

**Table 3:** Technological parameters of activation.

Binder	Furnace Time (h)	Temperature (°C)		Steam Pressure (Mpa)	Charge (kg/h)	The Obtained Number of Drums
		Charge	Discharge			
SBM	5	760–820	860–940	0.02–0.6	75	2340
SCMC I	4–5	520–760	720–1040	0.02–0.03	75	1476
SCMC II	5	600–670	960–1020	0.02–0.03	75	1944
SCMC + GT	4–5	500–720	740–1000	0.02	75	396
TO	5	620–690	940–1000	0.02	75	1332

Source: Own elaboration.

**Table 4:** Applied methodology for determination of binders and carbon parameters.

Lp.	Methodology No		Name
1.	PN-82	C-97555.00	Activated carbons. Methodology. General conditions and standard’s scope
2.	PN-82	C-97555.03	Activated carbons. Methodology. Determination of the methylene number
3.	PN-83	C-97555.04	Activated carbons. Methodology. Determination of iodine adsorption number
4.	PN-84	C-97555.05	Activated carbons. Methodology. Determination of the molasses number
5.	PN-83	C-97555.06	Activated carbons. Methodology. Determination of the phenol number
6.	PN-84	C-97555/07	Activated carbons. Methodology. Determination of the detergent number
7.	PN-84	C-97555/08	Activated carbons. Methodology. Determination of ash content
8.	PN-84	C-97555/09	Activated carbons. Methodology. Determination of water content
9.	PN-85	C-97555/10	Activated carbons. Methodology. Determination of the pH of an aqueous extract
10.	PN-86	C-97555/12	Activated carbons. Methodology. Determination of HCl-soluble substances
11.	PN-86	C-97555/14	Activated carbons. Methodology. Determination of the pH and Ca^2+^
12.	PN-88	C-97555/01	Activated carbons. Methodology. Mesh analysis
13.	PN-88	C-97555/11	Activated carbons. Methodology. Determination of water-soluble substances
14.	PN-80	G-04511	Solid fuels. Determination of moisture
15.	PN-80	G-04512	Solid fuels. Determination of ash content by weight
16.	PN	G-04516	Solid fuels. Determination of volatile parts content by weight
17.	PN-71	G-04501	Mesh analysis. Implementation guidelines
18.	PN-83	N-03010	Statistical quality control. Selection of random product to be sampled
19.	PN-81	C-04521/00	Determination of Fe. General conditions
20.	PN-81	C-04521.01	Determination of Fe with colorimetric method using batophenanthroline
21.	PN-81	C-04521/02	Determination of Fe with colorimetric method using 2,2′-dipiridil
22.	PN-81	C-04521.03	Determination of Fe with colorimetric method using ammonium thiocyanate
23.	PN-76	R-64772	Sugar beet molasses
24.	PN-ISO	2591-1	Mesh analysis. Methods using control screens from wire cloth and perforated metal sheet
25.	PN	C-04333	Coal-based products. Sampling and preparation of an average laboratory sample.
26.	PN	G-04502	Brown and hard coal. Sampling and preparation for laboratory tests. Basic methods
27.	PN-EN	12902	Products for drinking water treatment—Inorganic media and filtering materials. Test methods
28.	PN-EN	12915-1	Products for drinking water treatment—Granulated activated carbon. Part 1: Primary granular activated carbon
29.	PN-EN	12915-2	Products for drinking water treatment—Granulated activated carbon. Part 2: Reactivated granular activated carbon
30.	PN-ISO	1953	Hard coal. Grain analysis by sieving
31.	PN-ISO	9277	Determination of the specific surface area of solids by gas adsorption using the BET method

Source: Own elaboration.

**Table 5:** Properties of coal dust.

Binder	Content of				Apparent Surface (m^2^/g)	Grains Equivalent Ø (µm)
	Moisture (%)	Ash (%)	Volatile Substances (%)	Grains < 0.088 mm (%)		
SBM	2.4	5.5	31.00	95	0.27	21.9
SCMC I	2.74	5.95	30.85	97	0.31	18.24
SCMC II	1.48	4.35	29.14	97	0.27	12.18
SCMC + GT	3.15	4.77	29.66	97	0.22	26.79
TO	1.85	5.55	29.41	98.5	0.35	17.76

Source: own elaboration.

**Table 6:** Average properties of raw and dried granule samples.

Binder	Raw Granules	Dried Granules			
	Moisture (%)	Bulk Mass (g/dm^3^)	Moisture (%)	Volatile Matter (%)	Ash (%)
SBM	10.57	662	0.38-	33.2	6.34
SCMC I	18.44	593	0.47	30.89	5.95
SCMC II	15.94	590	0.57	31.15	3.9
SCMC + GT	19.87	550	1.41	32.31	6.39
TO	4.45	599	0.16	30.32	5.76

Source: own elaboration.

**Table 7:** Average properties of carbonized granules.

Indicator	SBM	SCMC I	SCMC II	SCMC + GT	TO
Bulk Mass (g/dm^3^)	603	590	612	592	653
Ash (%)	8.02	6.43	5.87	6.15	7.63
Volatile Matter (%)	18.23	20.13	8.49	26.43	9.03
Water Absorption (cm^3^/g)	0.36	0.30	0.38	0.37	0.34
Mechanical Strength (%)	91.40	78.00	98.60	78.90	99.70
Abrasion (%)	0.00	0.20	0.10	0.00	0.20
Granule Diameter (mm)	1.82	1.88	1.72	1.84	1.62
Granule Contraction (in Relation to Dried) (%)	5.99	0.53	7.02	4.66	12.90
Sieve Analysis—Sieve Residue (%):					
3.5 mm	1.20	0.60	0.70	0.60	0.40
2.75 mm	26.20	18.80	8.30	18.60	0.80
2.0 mm	60.80	64.80	67.10	67.80	31.40
1.5 mm	11.00	13.40	23.00	11.20	62.50
<1.0 mm	0.30	1.60	0.90	1.40	4.90
Porosimetry Tests—Pore Volume (cm^3^/g) for the Diameter Range:					
>7500 nm	0.01	0.03	0.03	0.01	0.01
7500–1500 nm	0.22	0.13	0.16	0.15	0.18
1500 mm–150 nm	0.10	0.14	0.11	0.15	0.06
150–15 nm	0.03	0.04	0.04	0.03	0.03
Total Pore Volume > 15 nm (cm^3^/g)	0.36	0.34	0.34	0.34	0.28

Source: own elaboration.

**Table 8:** Structural parameters of average GAC samples manufactured using the tested binders after the activation process.

Indicator	SBM			SCMC I			SCMC II			SCMC + GT			TO		
	1	2	3 and 4	1	2	3 and 4	1	2	3 and 4	1	2	3 and 4	1	2	3 and 4
Bulk Mass (g/dm^3^)	638	528	473	639	561	487	683	560	471	707	568	475	726	607	436
Ash (%)	11.09	14.03	17.34	10.34	12.50	14.75	7.68	9.83	12.31	9.10	13.10	17.58	8.86	10.88	17.82
Heat Efficiency (°C)	2.6	5.1	5.6	1.5	3.9	4.7	1.2	3.2	4.7	1.4	3.9	4.6	1.1	3.0	4.4
Water Absorption (cm^3^/g)	0.456	0.698	0.836	0.444	0.617	0.869	0.409	0.627	0.848	0.342	0.677	0.852	0.354	0.470	0.957
Dynamic Activity to C_6_H_6_ (Q C_6_H_6_) min.	21.6	51.9	57.2	10.2	37.3	51.4	14.0	38.0	51.1	12.3	40.0	42.3	10.2	30.9	51.9
Dynamic Activity to C_2_H_5_Cl (Q C_2_H_5_Cl) min.	18.3	39.6	40.7	15.7	31.5	31.5	15.9	30.3	36.3	15.2	33.0	29.7	12.0	27.7	31.6
Mechanical Strength (%)	100.0	96.5	92.5	98.5	96.9	90.0	100.0	97.5	95.7	96.9	95.0	89.2	99.7	97.0	86.5
Abrasion (%)	0.5	0.4	0.4	0.1	1.0	0.5	0.4	0.2	0.8	1.0	0.4	0.6	0.1	0.1	1.3
Granule Ø (mm)	1.61	1.54	1.45	1.64	1.59	1.49	1.59	1.52	1.40	1.64	1.48	1.41	1.48	1.42	1.4
Granule Contraction (compared to dry) (%)	17.0	20.6	25.2	13.2	15.9	21.2	14.0	17.8	24.3	15.0	23.3	26.9	20.4	23.7	24.7
Mesh Analysis (%):	–	–	–	–	–	–	–	–	–	–	–	–	–	–	–
3.5 mm	0.4	0.0	0.0	4.0	0.0	1.2	0.8	0.0	0.0	0.2	1.2	0.0	0.0	0.0	0
2.75 mm	3.2	0.7	0.1	4.8	0.6	0.3	2.6	1.0	0.1	1.2	0.2	0.1	0.6	0.1	0.1
2.0 mm	52.0	25.2	10.9	48.1	17.6	25.3	48.2	45.0	2.6	28.6	19.5	0.9	12.6	4.8	0.6
1.5 mm	43.2	67.0	79.0	40.6	68.8	63.8	45.2	44.6	79.2	58.8	65.9	70.2	77.0	86.2	66.7
1.0 mm	1.0	6.5	9.3	2.4	12.0	8.9	3.0	1.3	17.2	9.6	12.6	27.5	9.4	8.8	27.2
0.0 mm	0.2	0.6	0.7	0.1	1.0	0.5	0.2	0.1	0.9	1.6	0.6	1.3	0.4	0.1	5.4
Specific Surface BET (m^2^/g)	311.2	588.1	683.4	167.2	449.6	655.8	117.8	396.7	631.7	156.1	492.8	574.4	114.1	351.1	436
Porosimetry Tests—Pore Volume (cm^3^/g) for Ø:	–	–	–	–	–	–	–	–	–	–	–	–	–	–	–
>7500 nm	0.0244	0.0209	0.0247	0.0448	0.0378	0.0414	0.4140	0.0428	0.0362	0.0285	0.0295	0.0521	0.0243	0.0216	0.0306
7500–1500 nm	0.1895	0.2146	0.2413	0.1249	0.1574	0.1533	0.1442	0.1659	0.2331	0.1064	0.1282	0.2222	0.1697	0.1766	0.2591
1500 mm–150 nm	0.1235	0.1668	0.2135	0.1305	0.1475	0.2008	0.0908	0.1139	0.1358	0.1064	0.1451	0.1522	0.0662	0.0917	0.1801
150–15 nm	0.0250	0.0478	0.0748	0.0343	0.0729	0.1115	0.0341	0.0842	0.131	0.0284	0.0622	0.1221	0.0324	0.0608	0.1896

Source: own elaboration.

**Table 9:** Average properties of final GAC granules with different binders.

Binder	Bulk Mass (g/dm^3^)	Water Absorption (cm^3^/g)	Mechanical Strength (%)	Ash Content (%)	Q C_6_H_6_ Minimum	Q C_2_H_5_Cl Minimum	Granule Ø (mm)	BET (m^2^/g)
SBM	422	0.856	95.1	3.95	61.4	43.8	1.45	769.4
SCMC I	435	0.888	89.8	3.69	51.4	33.9	1.49	718.3
SCMC II	451	0.854	96.1	3.58	53.1	34.9	1.40	698.9
SCMC + GT	433	0.890	87.0	4.39	48.9	30.5	1.41	637.1
TO	426	0.954	85.7	4.46	48.0	30.7	1.40	501.3

Source: own elaboration.

## Supplementary Materials

The following are available online at https://www.mdpi.com/1996-1944/13/22/5180/s1, Figure S1: Stone mill for hard coal grinding, Figure S2: Coal and binder paste, Figure S3: Granulator output, Figure S4: Activation furnace, Table S1: Analytical methods and procedures used for individual active coal’s indicators determination.
