# Supplementary material for: Research on the Development of Technologies for the Production of Granulated Activated Carbons Using Various Binders
Source: Materials (Basel). 2020 Nov 17;13(22):5180. doi: 10.3390/ma13225180 (PMC7698345; doi:10.3390/ma13225180)
Supplement: Supplementary file 1 [file materials-13-05180-s001.pdf]

materials

*Supplementary Matrials*

# Research on the Development of Technologies for the Production of Granulated Activated Carbons Using Various Binders†

**Iwona Skoczko * and Remigiusz Guminski**

**Table 1.** Analytical methods and procedures used for individual active coal's indicators determination.

| | Parameter | Method |
|---|---|---|
| 1 | Volatile Matter [%] | The method of determination of volatile parts consists in roasting in an electric furnace the weight of 1 ± 0.1 g analytical sample of solid fuel, in a closed porcelain pot with standardized dimensions, without air, at 850 ± 15 °C for 7 min and then calculation of the volatile parts content, as the difference between the total weight loss, and the loss caused by the loss of moisture. |
| 2 | Ash Content [%] | Determination of ash content consists of complete combustion and annealing in a muffle furnace heated to 1088 ± 10 K (815 ± 10 °C), an analytical sample of coal with a mass of 1 g ± 0.1 crushed to grains below 0.2 mm. The sample is gradually introduced into the heated muffle furnace and then, after the furnace door is closed, oxygen is introduced into it. Ashing under these conditions takes 10 min. |
| 3 | Moisture [%] | The moisture is determined by the drying method, by drying the air-dry coal sample in an air atmosphere at a temperature of 378–383 K (105–110 °C), in an electric dryer with thermostatic temperature control. For the determination, a quantity of approximately 1 g is used, for grains below 0.2 mm (analytical test). Drying is run for 60 min for hard coal, then the glass vessel with it is closed with a lid, removed from the dryer and places it in a desiccator and then, when cooled, weighs on an analytical scale. Drying is repeated at 15-min intervals as long as the mass is not determined with an accuracy of ±0,0010 g. |
| 4 | Bulk Mass [g/dm$^3$] | It is a quantity characterizing the ratio of the total mass of grains, granular or porous material to the volume it occupies. It consists in determining the volume of material of known mass. |
| 5 | Abrasion [%] | Abrasion is a mechanical property of a material, which is determined by measuring the loss of height or weight per cm$^2$ of the surface area of the sample subjected to abrasion. Carbon abrasion is determined as loss of mass during 15 min of shaking on a sieve and presented as a ratio of the sample mass before and after shaking.. |
| 6 | Mechanical Strength [%] | The method consists in placing a screened and weighed carbon sample together with stainless steel balls (15 balls with a diameter of 12.7 +/−0.1 mm and 15 balls with a diameter of 9.5 +/−0.1 mm) on a special testing pan, then subjecting it to vibrations and rotation for 30 min. (mechanical shaker producing 140–160 vibrations and 280–320 rotational movements per minute in a set with standard 200 mm diameter and 50 mm high screens). Particle size degradation is determined by measuring the mass of carbon remaining on the sieve with the mesh size closest to half of the mesh size of the sieve determining the minimum diameter of the primary sample particles. |
| 7 | Water Absorption [cm$^3$/g] | Performing the determination, the weight of carbon should be transferred to a conical flask and water should be poured into it, then the flask with its contents should be heated to the boil and maintained for 15 min. Then whole mixture ought to be cooled and connected the set with the vacuum pump and the Buchener funnel. Drain the water from the carbon on the funnel occurs at a vacuum of 60–65 mm Hg. The suction time should be 5 min. After this time, water absorption is measured trough comparison a weigh of the coal mass with a sample soaked in water. |
| 8 | BET [m$^2$/g] | The measurements of the specific surface of the samples were made with the use of a sorption apparatus by adsorption of high-purity nitrogen at liquid nitrogen temperature. Before the measurements, the samples were dried in a vacuum at a temperature of about 105 °C to obtain a constant mass. The state of surface degassing was controlled and completely desorbed samples were used for measurement. The weighed sample was poured into a burette, which was tightly closed and connected to a vacuum system at 3 × 10$^{-2}$ mbar. The burette was then connected to a Dewar vessel containing liquid nitrogen. The time delay was 1 min to reach full degassing sample and pressure was below 5 Pa. Such conditions allowed the burette to open and read the result on the apparatus the specific surface area BET (in m$^2$/g). |

Source: Elaboration of active coal parameters measurement by industrial plant producing GAC for experiments.

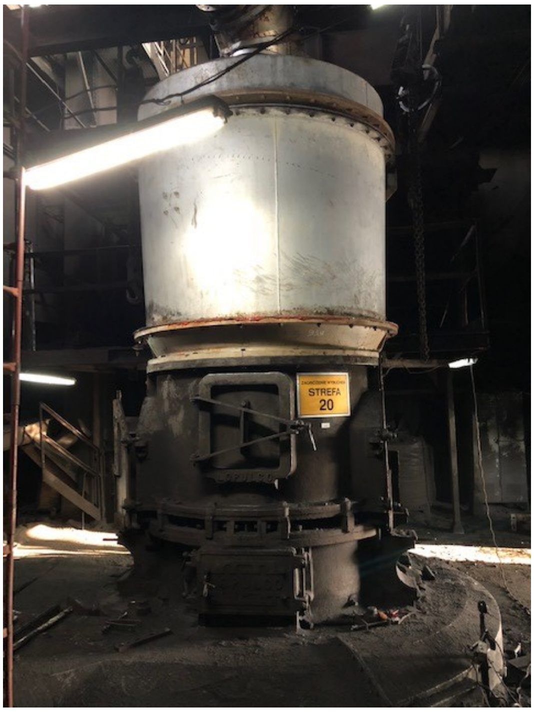


**Figure S1.** Stone mill for hard coal grinding (own elaboration).

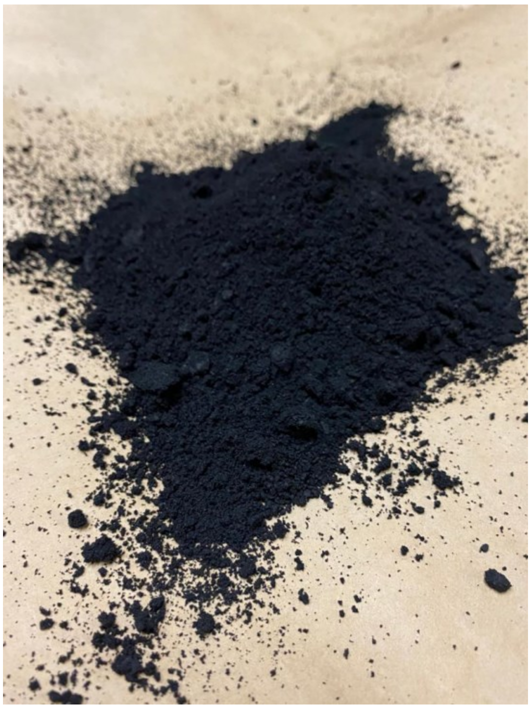

**Figure S2.** Coal and binder paste (own elaboration).

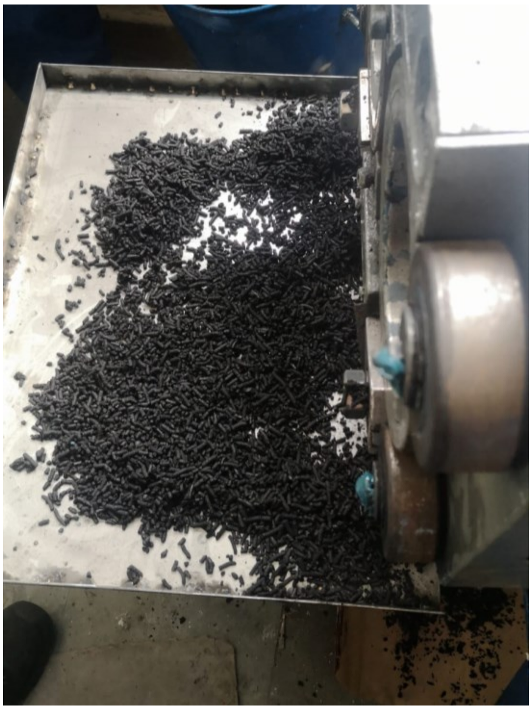

**Figure S3.** Granulator output (own elaboration).

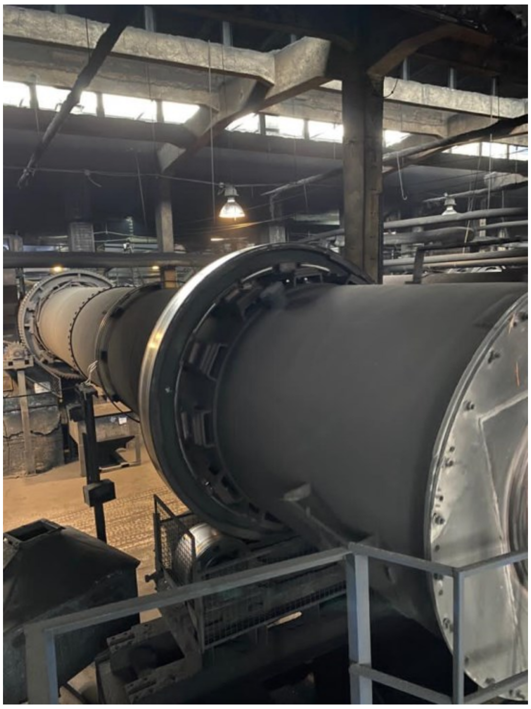

**Figure S4.** Activation furnace (own elaboration).
